# RNA sequencing data of mouse 2-cell embryos treated with DMSO

**DOI:** 10.1016/j.dib.2019.105025

**Published:** 2019-12-19

**Authors:** Min-Hee Kang, Seong-Yeob You, Kwonho Hong, Jin-Hoi Kim

**Affiliations:** Department of Stem Cell and Regenerative Biotechnology, Humanized Pig Research Center (SRC), Konkuk University, Seoul, Republic of Korea

**Keywords:** Dimethyl sulfoxide, RNA sequencing, Preimplantation embryo, Epigenetics, Acetylation

## Abstract

To understand the effect of DMSO in preimplantation embryos, we have treated mouse 1 cell zygotes with DMSO and found that DMSO treatment caused 2 or 4 cell embryonic arrest and altered the acetylation levels of mouse preimplantation embryos To illustrate the mechanism of DMSO in mouse preimplantation embryos, fertilized zygotes have been treated with 2% of DMSO and then performed RNA-seq analyses. Differentially expressed genes were identified using DESeq2 after adjustment for false discovery rate (FDR q value < 0.05). Gene Set Enrichment Analysis (GSEA) was also performed to identify biological pathways significantly modulated by DMSO. Raw and processed RNA-seq data were deposited and made publicly available on the Gene Expression Omnibus (GEO; GSE124598). The data presented in this article are related to the research paper entitled “DMSO impairs the transcriptional program for maternal-to-embryonic transition by altering histone acetylation”, available in Biomaterials [1].

**Table:** Specifications Table

Subject	Developmental Biology
Specific subject area	Molecular biology of mouse embryos; Epigenetics; Genomic activation
Type of data	Figures, Table
How data were acquired	High-throughput sequencing using Illumina HiSeq2500 and computational working in R software.
Data format	- Raw data in repository: mapped reads data (.bedgraph) and calculated TPM values for each gene (.txt). - Statistically analysed and filtered differentially expressed genes (DEGs), gene ontology (GO), and pathways (supplementary data;.xls).
Parameters for data collection	Two groups of 2-cell embryos were used. One group is treated with 2% DMSO and another group is control.
Description of data collection	We cultured 18 hours post hCG zygotes in KSOM media supplemented with or without 2% DMSO for 24 hours and then fifty numbers of developed 2-cell embryos in each group were subjected to low-put RNA sequencing. Raw FASTQ files were mapped and quantified using Kallisto tool and differentially expressed genes (DEGs) were analyzed by DESeq2 package in R. Also, enrichment tests based on KEGG and REACTOME pathways for DEGs were conducted using ClueGO and CluePedia plug-in in Cytoscape 3.6 software.
Data source location	Konkuk University, Seoul, South Korea
Data accessibility	Repository name: Gene Expression Omnibus (GEO) Data identification number: GSE124598 Direct URL to data: https://www.ncbi.nlm.nih.gov/geo/query/acc.cgi?acc=GSE124598
Related research article	Author's name: Min-Hee Kang, Seong-Yeob You, Kwonho Hong, and Jin-Hoi Kim Title: DMSO impairs the transcriptional program for maternal-to-embryonic transition by altering histone acetylation Journal: Biomaterials https://doi.org/10.1016/j.biomaterials.2019.119604

**Table:** 

**Value of the Data** • The network data analysis such as gene ontology (GO), molecular pathways, and transcriptomic analysis of 2-cell embryos treated with DMSO could provide novel insights about the differential responses between maternal and embryonic clock. • Mapped reads data and TPM values in raw data could be useful to predict developmental arrest of early embryos via incomplete epigenetic reprogramming and cellular stress induced by DMSO. • RNA-seq analysis offer researchers to test whether DMSO is associated with possible toxicity and/or a range of serious side effects in cellular function and growth. • Mouse preimplantation embryo-based assays can provide timely alerts about widespread applications of DMSO as a positive control or drug solvent agent.

## 1 Data

**Fig. 1:**
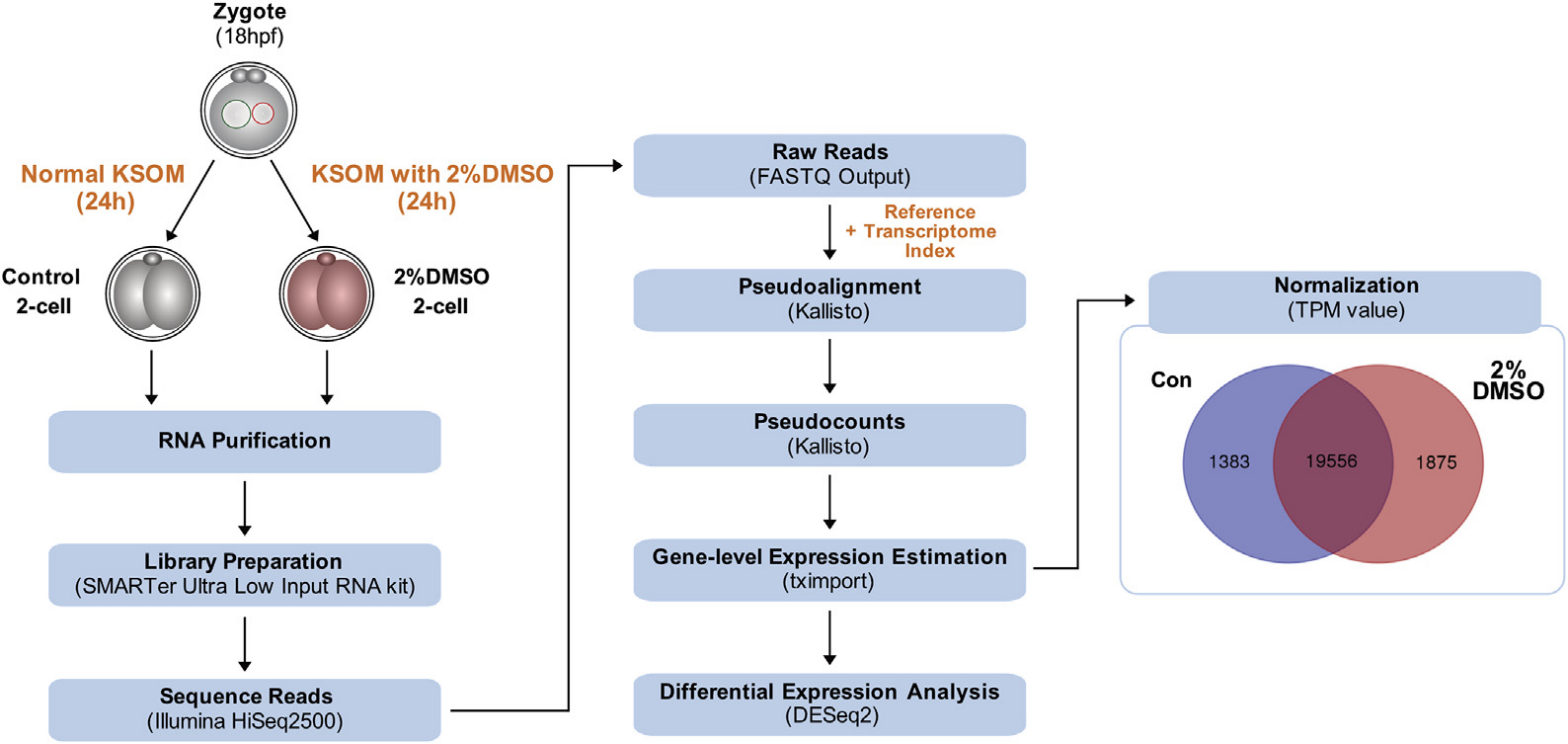
**Pipeline of RNA-Seq analysis for DMSO-treated 2-cells.** Based on gene-level expression estimation, 19,556 genes were expressed in common in both control and treated groups.

Datasets presented here were employed in the main work “DMSO impairs the transcriptional program for maternal-to-embryonic transition by altering histone acetylation” Kang et al., 2020 [1]. Fig. 1 illustrates the experimental procedure. RNA-seq analysis was performed in 2-cell mouse embryos cultured after supplementation of 2% DMSO. The raw data generated from Illumina sequencing were deposited on the Gene Expression Omnibus (GEO) with the reference number GSE124598 (https://www.ncbi.nlm.nih.gov/geo/query/acc.cgi?acc=GSE124598).

**Fig. 2:**
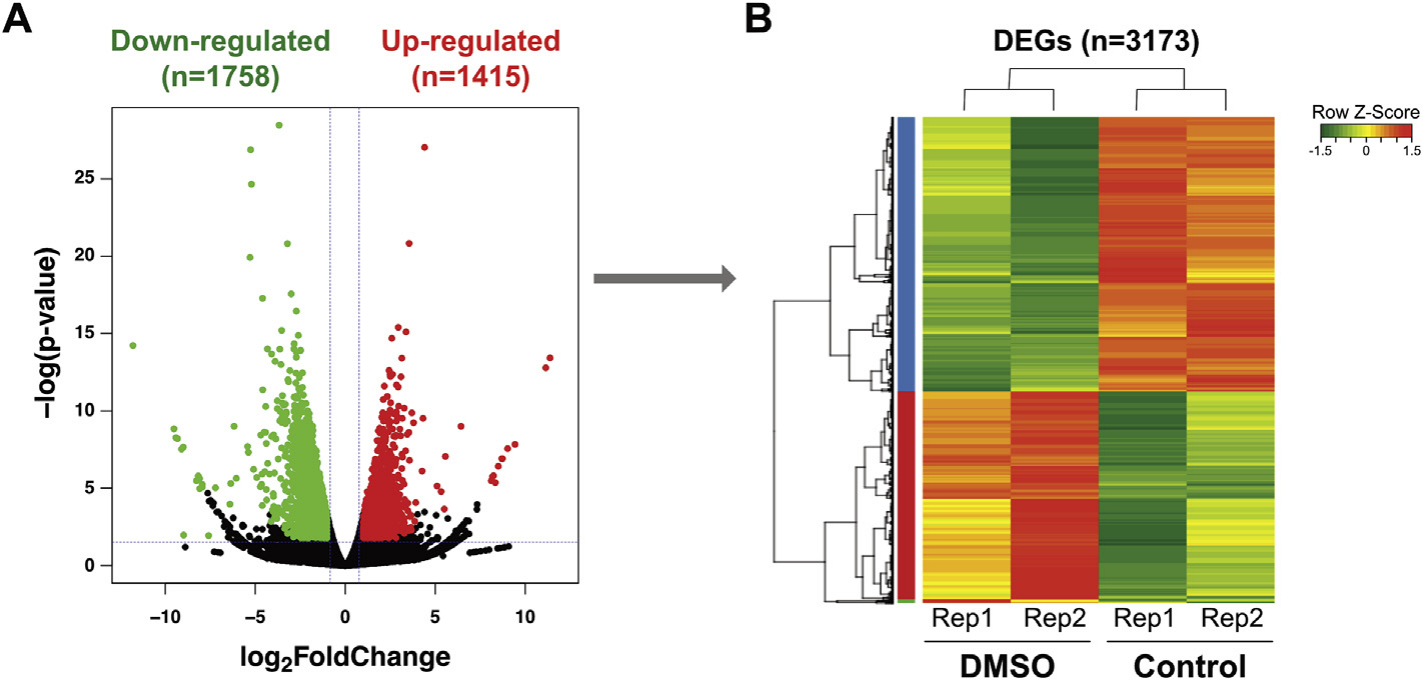
**Up- and Down-regulated differentially expressed genes (DEGs) by DMSO in 2-cell embryos.** (A) Each DEG is plotted with logged p-value and fold change values as scatter plot. Up- and down-regulated genes are represented as red and green dots, respectively (|fold change| >2; *p*-value < 0.05). (B) Significantly changed DEGs (n = 3,173) were hierarchically clustered with heatmap based on logged TPM value. Detailed DEGs and TPM values are listed in supplementary data and data repository (https://www.ncbi.nlm.nih.gov/geo/query/acc.cgi?acc=GSE124598).

**Fig. 3:**
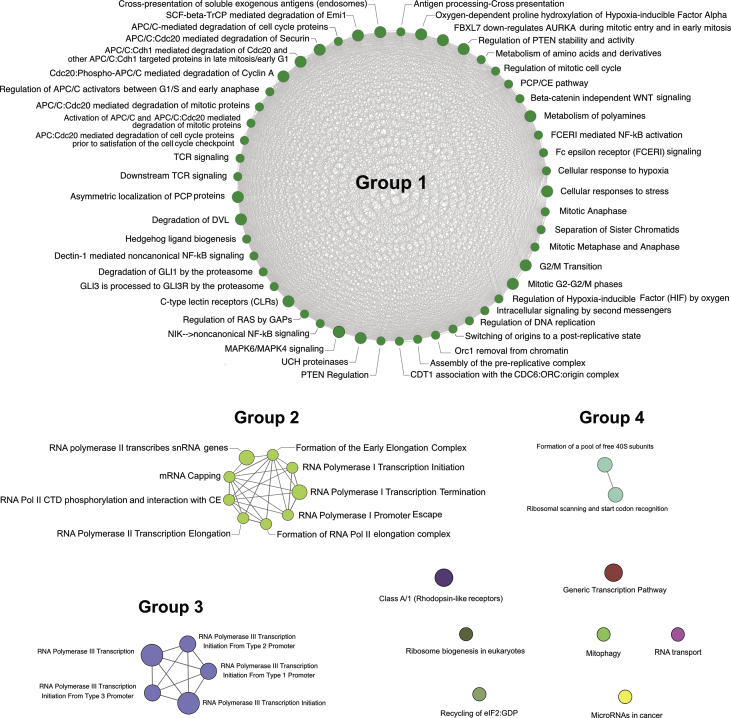
**Interactive string network of KEGG and REACTOME pathways for DEGs.** Enriched KEGG and REACTOME pathways for DEGs are mainly clustered as Group 1–4 using ClueGO plug-in in Cytoscape 3.6. Detailed genes on each pathway node are listed in Table 1 and Supplementary Data.

RNA-seq analysis was performed in the 2-cell embryos with/without DMSO supplementation. In total, 3,742, which is ∼20.29% of the total valid genes, genes were differentially expressed in DMSO-treated embryo compared with control embryo with criteria of FDR < 0.05. Of these differentially expressed genes, 1,415 genes were up-regulated, whereas 1,758 genes were down-regulated in DMSO-treated embryo (Fig. 2). DEGs were significantly enriched in total 72 KEGG and REACTOME pathways terms (adjusted p-value < 0.01) and the terms were mainly clustered into 4 characterized groups (Fig. 3).

**Fig. 4:**
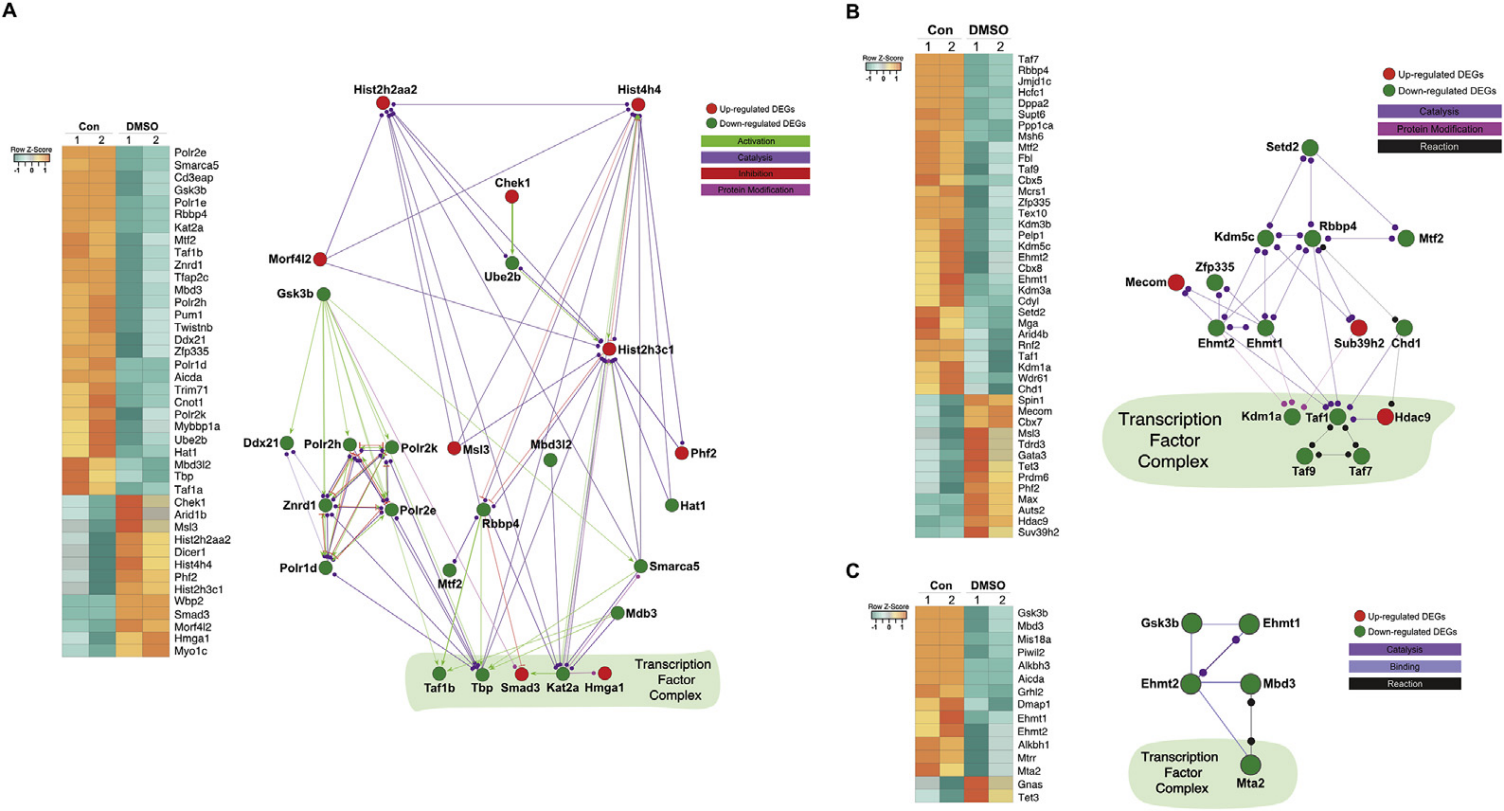
**Interrelation network among enriched DEGs for epigenetic gene expression and histone modification.** Based on GO enrichment test by ClueGO, heat maps and pathway-like visualizations for DEGs that associated with epigenetic gene expression (A), histone methylation (B) and histone acetylation (C) were created using CluePedia plug-in in Cytoscape 3.6 software. Functional relations between DEGs were drawn by colored lines, which represent activation (green), catalysis (purple), inhibition (red), protein modification (light purple) and reaction (black).

Next, we interpreted potential interactive pathways among DEGs associated with epigenetic gene expression, histone modifications (acetylation and methylation) in DMSO treated group using cerebral layout (Fig. 4). Most of DEGs for histone modifications and binding events are significantly depressed at specific and highly characteristic genomic elements and locations in DMSO-treated groups, indicating that DMSO exhibits specific regulatory mechanisms related to regulation of transcription factors, compared with control embryos.

In this study, we proved our hypothesis by RNA-seq analysis to monitor the early embryonic impacts after exposure to DMSO and identified previously unknown underlying molecular mechanisms that explain the DMSO-induced embryonic toxicity, embryo loss, and infertility. Our study suggests for the first time that DMSO exposure induces a significant alteration in gene expression and the functionality of preimplantation embryos via alternations in epigenetic reprogramming. Thus, our findings emphasize that the use of DMSO as a standard control test or solvent requires far more cautious consideration, because DMSO can alter cell function by acting as a proteasome or HDAC inhibitor as well as inducing cell toxicity.

**Table 1:** KEGG and REACTOME pathway analysis in DMSO-treated 2-cell embryos.

	Pathway ID	Pathway Term	adj_pvalue	No. of Genes	% Genes
1	R-MMU:2262752	Cellular responses to stress	0.00107233	92	24.02
2	R-MMU:72702	Ribosomal scanning and start codon recognition	0.00109672	23	41.07
3	R-MMU:1234176	Oxygen-dependent proline hydroxylation of Hyposia-inducible factor alpha	0.00115378	24	40.00
4	R-MMU:72689	Formation of a pool of free 40S subunits	0.00117991	20	44.44
5	R-MMU:8948751	Regulation of PTEN stability and activity	0.00118317	25	39.06
6	R-MMU:373076	Class A/1 (Rhodopsin-like receptors)	0.00134226	20	6.25
7	R-MMU:4641258	Degradation of DVL	0.00147675	22	41.51
8	R-MMU:74158	RNA Polymerase III Transcription	0.00154192	17	48.57
9	R-MMU:76046	RNA Polymerase III Transcription Initiation	0.00154192	17	48.57
10	R-MMU:6807505	RNA polymerase II transcribes snRNA genes	0.00160451	27	36.99
11	R-MMU:69275	G2/M Transition	0.00170795	50	28.57
12	R-MMU:212436	Generic Transcription Pathway	0.00209786	135	21.63
13	R-MMU:453274	Mitotic G2-G2/M phases	0.00214072	50	28.25
14	R-MMU:4608870	Asymmetric localization of PCP proteins	0.00222022	23	39.66
15	R-MMU:5689603	UCH proteinases	0.00278995	32	33.33
16	R-MMU:174113	SCF-beta-TrCP mediated degradation of Emi1	0.00288664	21	41.18
17	R-MMU:8854050	FBXL7 down-regulates AURKA during mitotic entry and in early mitosis	0.00288664	21	41.18
18	R-MMU:5687128	MAPK6/MAPK4 signaling	0.00300873	26	36.62
19	R-MMU:174154	APC/C:Cdc20 mediated degradation of Securin	0.00312854	24	38.10
20	R-MMU:5621481	C-type lectin receptors (CLRs)	0.003421	35	31.82
21	R-MMU:73863	RNA Polymerase I Transcription Termination	0.00380234	15	50.00
22	R-MMU:1236978	Cross-presentation of soluble exogenous antigens (endosomes)	0.00388676	20	41.67
23	R-MMU:174178	APC/C:Cdh1 mediated degradation of Cdc20 and other APC/C:Cdh1 targeted proteins in late mitosis/early G1	0.00417739	25	36.76
24	R-MMU:174184	Cdc20:Phospho-APC/C mediated degradation of Cyclin A	0.00417739	25	36.76
25	R-MMU:351202	Metabolism of polyamines	0.0042154	29	34.52
26	R-MMU:68882	Mitotic Anaphase	0.00531307	52	27.08
27	KEGG:03008	Ribosome biogenesis in eukaryotes	0.00545287	36	31.03
28	R-MMU:179419	APC:Cdc20 mediated degradation of cell cycle proteins prior to satisfaction of the cell cycle checkpoint	0.00560289	25	36.23
29	R-MMU:1234174	Regulation of Hypoxia-inducible Factor (HIF) by oxygen	0.00579829	24	36.92
30	R-MMU:2262749	Cellular response to hypoxia	0.00579829	24	36.92
31	R-MMU:5610780	Degradation of GLI1 by the proteasome	0.00584049	21	39.62
32	R-MMU:72086	mRNA Capping	0.00770714	14	50.00
33	R-MMU:112382	Formation of RNA Pol II elongation complex	0.00820743	21	38.89
34	R-MMU:75955	RNA Polymerase II Transcription Elongation	0.00820743	21	38.89
35	R-MMU:2555396	Mitotic Metaphase and Anaphase	0.00834028	52	26.94
36	R-MMU:6807070	PTEN Regulation	0.00889033	34	31.48
37	R-MMU:3858494	Beta-catenin independent WNT signaling	0.01005271	37	30.08
38	R-MMU:2871837	FCERI mediated NF-kB activation	0.01119674	26	35.14
39	R-MMU:5358346	Hedgehog ligand biogenesis	0.01121237	22	37.29
40	R-MMU:5607761	Dectin-1 mediated noncanonical NF-kB signaling	0.01133035	21	38.18
41	R-MMU:5610785	GLI3 is processed to GLI3R by the proteasome	0.01133035	21	38.18
42	R-MMU:5676590	NIK-->noncanonical NF-kB signaling	0.01133035	21	38.18
43	R-MMU:68827	CDT1 association with the CDC6:ORC:origin complex	0.01133035	21	38.18
44	R-MMU:73772	RNA Polymerase I Promoter Escape	0.01271169	14	48.28
45	KEGG:03013	RNA transport	0.01396122	46	27.54
46	R-MMU:2454202	Fc epsilon receptor (FCERI) signaling	0.01424613	36	30.00
47	R-MMU:5658442	Regulation of RAS by GAPs	0.01448249	23	35.94
48	R-MMU:68867	Assembly of the pre-replicative complex	0.01448249	23	35.94
49	R-MMU:73762	RNA Polymerase I Transcription Initiation	0.01479977	18	40.91
50	R-MMU:5205647	Mitophagy	0.01554993	13	50.00
51	R-MMU:77075	RNA Pol II CTD phosphorylation and interaction with CE	0.01554993	13	50.00
52	R-MMU:176409	APC/C:Cdc20 mediated degradation of mitotic proteins	0.01645248	25	35.21
53	R-MMU:176814	Activation of APC/C and APC/C:Cdc20 mediated degradation of mitotic proteins	0.01850655	25	34.72
54	R-MMU:113418	Formation of the Early Elongation Complex	0.0203327	14	46.67
55	R-MMU:9006925	Intracellular signaling by second messengers	0.02051725	62	24.60
56	R-MMU:2467813	Separation of Sister Chromatids	0.02055007	48	26.52
57	R-MMU:176408	Regulation of APC/C activators between G1/S and early anaphase	0.02193502	26	33.33
58	KEGG:05206	MicroRNAs in cancer	0.02406351	19	6.76
59	R-MMU:76061	RNA Polymerase III Transcription Initiation From Type 1 Promoter	0.02559861	13	48.15
60	R-MMU:76066	RNA Polymerase III Transcription Initiation From Type 2 Promoter	0.02559861	13	48.15
61	R-MMU:202424	Downstream TCR signaling	0.02570574	28	32.56
62	R-MMU:72731	Recycling of eIF2:GDP	0.02607639	7	77.78
63	R-MMU:71291	Metabolism of amino acids and derivatives	0.0304537	60	24.69
64	R-MMU:76071	RNA Polymerase III Transcription Initiation From Type 3 Promoter	0.04105632	13	46.43
65	R-MMU:202403	TCR signaling	0.04107335	31	30.10
66	R-MMU:174143	APC/C-mediated degradation of cell cycle proteins	0.04146255	27	32.14
67	R-MMU:453276	Regulation of mitotic cell cycle	0.04146255	27	32.14
68	R-MMU:69304	Regulation of DNA replication	0.04440004	24	33.33
69	R-MMU:68949	Orc1 removal from chromatin	0.04507583	23	34.33
70	R-MMU:69052	Switching of origins to a post-replicative state	0.04507583	23	34.33
71	R-MMU:1236975	Antigen processing-Cross presentation	0.04695947	27	31.76
72	R-MMU:4086400	PCP/CE pathway	0.04695947	27	31.76

## 2 Experimental design, materials, and methods

### 2.1 Animals and embryo collection

BDF1 (C57BL/6 × DBA/2; F1; Orient Bio Co. Ltd) mice (8–12 weeks olds) were used for analysis according to guidelines approved by the committee on animal care and use at Konkuk University (IACUC approval number: KU18199). Intraperitoneally injection was carried out in female mice were with pregnant mare's serum gonadotropin (PMSG; G4527, Sigma Aldrich; 5IU) followed human chorionic gonadotropin (hCG; CG10, Sigma Aldrich; 5IU) 48 h later, then mated with male mice. Fertilized oocytes with two pronuclei were collected from oviduct at 18–20 h of post hCG injection and each 10 zygotes was cultured in 20ul KSOM (95mM NaCl, 2.5mM KCl, 0.35mM KH_2_PO_4_, 0.2mM MgSO_4_, 10mM Sodium Lactate, 0.2mM Glucose, 0.2mM Sodium pyruvate, 25mM NaHCO_3_, 1mM Glutamine, 0.01mM Ethylenediaminetetraacetic acid, 5mg/ml Bovine albumine serum) supplemented with 2% DMSO (D2650, Sigma Aldrich) or without. BDF1 embryos with second polar body were collected and cultured in KSOM with/without 2% DMSO for further analysis.

### 2.2 Library preparation and RNA-seq

Fifty 2-cell embryos from each control and DMSO-treated group were directly subjected to cDNA synthesis using SMARTer® Ultra® Low Input RNA Kit (634940, Clonetech) according to the manufacturer's instructions. RNA quality was determined using the Agilent Bioanalyzer High Sensitivity DNA kit (5067-4626, Agilent). The synthesized cDNAs with 150-200bp size were used for the preparation of sequencing library using Low Input DNA Library Prep Kit (634946, Clonetech) according to the manufacturer's instructions, and subjected to size selection, followed paired-end reads data were obtained by performing 50 bp sequencing using HiSeq2500 (Illumina).

### 2.3 RNA-seq data analysis

Reads were pseudomapped using kallisto [2] with default parameters by transcriptome index from FASTA formatted transcriptomes files (GRCm38.re179) of ENSEMBL transcript database (mm10). Transcript abundance of each gene was quantified with the parameters (quant -t -b 100) as transcripts per kilobase million (TPM) using kallisto. Gene-scaled TPM values for each gene transcript were summed by tximport [3] in R/Bioconductor. Differentially expressed gene (DEG) were analyzed by DESeq2 [4] in R/Bioconductor with the parameters (baseMean counts >14; false discovery rate (FDR) < 0.05).

### 2.4 Pathway enrichment test and *in silico* analysis

DEGs were tested for pathway enrichment score in KEGG and REACTOME pathways using ClueGO [5] plug-in in Cytoscape 3.6 (http://www.cytoscape.org). To search potential associations among DEGs specific gene ontology (GO) terms regarding epigenetic gene expression, histone acetylation and histone methylation, ClueGO enrichment test were integrated into CluePedia [6] plug-in in Cytoscape 3.6 and analyzed.
